# Dracorhodin perchlorate induces the apoptosis of glioma cells

**DOI:** 10.3892/or.2021.8049

**Published:** 2021-04-12

**Authors:** Xin Chen, Junjie Luo, Linghu Meng, Taifeng Pan, Binjie Zhao, Zhen-Gang Tang, Yongjian Dai

Oncol Rep 35: 2364-2372, 2016; DOI: 10.3892/or.2016.4612

Following the publication of the above paper, a concerned reader drew to the Editors attention that certain of the western blotting data appeared to have been duplicated, comparing Fig. 2B with Fig. 4A; furthermore, the flow cytometric data panels featured in Fig. 3A appeared to contain repeated patternings of data within those data panels.

After having conducted an independent investigation in the Editorial Office, the Editor of *Oncology Reports* has determined that this paper should be retracted from the Journal on account of a lack of confidence concerning the originality and the authenticity of the data. The authors were asked for an explanation to account for these concerns, but the Editorial Office never received any reply. The Editor regrets any inconvenience that has been caused to the readership of the Journal.

